# Supplementary material for: Lowering the barriers to sexual health services: Impacts of free counselling and testing for sexually transmitted infections in Switzerland – an observational study
Source: PLoS One. 2026 Apr 1;21(4):e0327114. doi: 10.1371/journal.pone.0327114 (PMC13042815; doi:10.1371/journal.pone.0327114)
Supplement: S1 File — (PDF) [file pone.0327114.s001.pdf]

S1 Supporting Information: Description of the laboratory tests performed for STI / HIV diagnosis

HIV: enzyme-linked immunosorbent assays (ELISA) or 4th generation rapid tests

Syphilis: chemiluminescent microparticle immunoassay (CMIA) for people with no history of previous syphilis infection and venereal disease research laboratory test (VDRL)/rapid plasma regain test (RPR) for people with a history of previous syphilis infection

Chlamydia and gonorrhoea: both polymerase chain reaction (PCR) with pooled swabs (pharyngal, urethral/vaginal and anal

Hepatitis C: qualitative testing (Anti-HCV ql)
