# Supplementary material for: Lowering the barriers to sexual health services: Impacts of free counselling and testing for sexually transmitted infections in Switzerland – an observational study
Source: PLoS One. 2026 Apr 1;21(4):e0327114. doi: 10.1371/journal.pone.0327114 (PMC13042815; doi:10.1371/journal.pone.0327114)
Supplement: S2 File — (PDF) [file pone.0327114.s002.pdf]

Supporting Information S2: Description of the data sets

BerDa data: VCT in the general population

The Swiss Federal Office of Public Health (FOPH) has developed an online tool BerDa ("Beratungsleitfaden und Datenverwaltungssystem") for VCT centres. This tool administers an anonymous, digital questionnaire prior to the counselling to gather self-reported socio-behavioural data from the clients (e.g., year of birth, sexual orientation, timepoint and result of last HIV test, sexual partners), integrates data from the VCT counsellor (e.g., STI testing and results), and provides operational data (e.g., number of visits). Clients are assigned a unique BerDa-ID, which they are encouraged to use during future visits. However, because the data is collected anonymously, some clients may visit multiple times without using the same ID, making it difficult to accurately track repeat visits.

SwissPrEPared data: VCT in the population eligible for PrEP

The SwissPrEPared tool was developed specifically to provide integrated medical care and data collection for individuals taking PrEP in Switzerland. More details on its development and programmatic and research-related uses have been published elsewhere [1–5]. Similarly to the BerDa data, this tool administers a digital questionnaire prior to the VCT appointment to gather self-reported socio-behavioural data from the clients to guide the VCT and/or PrEP appointment (e.g., number of sexual partners, mental health), integrates data from the VCT counsellor (e.g., STI testing and results), and provides operational data (e.g., number of visits). These data were used for participants that received PrEP counselling as part of their visits.

Clients' feedback questionnaire (FBQ)

One week after their visit, VCT clients receive a text message link to an anonymous digital online feedback questionnaire (FBQ). Importantly, the FBQ is not sent to SwissPrEPared participants (i.e., those receiving PrEP counselling) as they are part of routine follow-up for their PrEP care. The FBQ includes questions about their sociodemographic background, experiences during the VCT and with sexual

healthcare in general, and the barriers they have experienced to obtaining STI testing. Participants could select one or more of the following as a barrier: (1) money, (2) no knowledge of places which test, (3) embarrassment, (4) time, (5) fearing reaction of friends/relatives, (6) fear of results, (7) unmotivated, (8) kept by no concrete hurdle. The FBQ cannot be linked with BerDa data due to its anonymous nature.

References

1. Hampel B, Farnham A, Lamothe-Molina PJ, Capelli C, Schibler M, Alonso PU, et al. Low prevalence of asymptomatic mpox in populations at high risk. Lancet Microbe [Internet]. 2023 Aug 16 [cited 2023 Sep 29];0(0). Available from: https://www.thelancet.com/journals/lanmic/article/PIIS2666-5247(23)00248-3/fulltext
2. Hovaguimian F, Martin E, Reinacher M, Rasi M, Schmidt AJ, Bernasconi E, et al. Participation, retention and uptake in a multicentre pre-exposure prophylaxis cohort using online, smartphone-compatible data collection. HIV Med. 2022 Feb;23(2):146–58.
3. Farnham A, Frei A, Kopp J, Schori LJ, Kotoun OJ, Reinacher M, et al. Sexual behaviours among MSM during the first COVID-19 lockdown not associated with risk of COVID-19 infection. BMC Public Health. 2024;24: 3038. doi:10.1186/s12889-024-20514-9
4. Winter BL, Hovaguimian F, Kouyos RD, Schmidt AJ, Bernasconi E, Braun DL, et al. Changes in mental and sexual health among MSM using HIV pre-exposure prophylaxis during the SARS-CoV-2 pandemic: longitudinal analysis of the SwissPrEPared cohort study. Swiss Med Wkly. 2022 Jun 27;152(2526):w30192–w30192.
5. Hovaguimian F, Kouyos RD, Kusejko K, Schmidt AJ, Tarr PE, Bernasconi E, et al. Incidence of sexually transmitted infections and association with behavioural factors: Time-to-event analysis of a large pre-exposure prophylaxis (PrEP) cohort. HIV Med. 2024;25(1):117–28.
