# Supplementary material for: Lowering the barriers to sexual health services: Impacts of free counselling and testing for sexually transmitted infections in Switzerland – an observational study
Source: PLoS One. 2026 Apr 1;21(4):e0327114. doi: 10.1371/journal.pone.0327114 (PMC13042815; doi:10.1371/journal.pone.0327114)
Supplement: S3 File — (PDF) [file pone.0327114.s003.pdf]

S3 Supporting Information: Definitions of demographic and sexual identity groups

Men having sex with men (MSM): Male gender and either homosexual, bisexual or pansexual

Men having exclusively sex with women (MSW): Male gender and heterosexual

Women having sex with men (WSM): Female gender and either heterosexual, bisexual or pansexual

Women having exclusively sex with women (WSW): Female gender and homosexual

Gender-diverse: Either non-binary or using other term to describe their gender
