# Supplementary material for: Lowering the barriers to sexual health services: Impacts of free counselling and testing for sexually transmitted infections in Switzerland – an observational study
Source: PLoS One. 2026 Apr 1;21(4):e0327114. doi: 10.1371/journal.pone.0327114 (PMC13042815; doi:10.1371/journal.pone.0327114)
Supplement: S5 Table — 1Entries with age provided, CH-Li: Switzerland or Lichtenstein. (PDF) [file pone.0327114.s005.pdf]

S5 Table: Descriptives of people < 26 years of age and people on low incomes filling in the feedback questionnaire (FBQ)

N_{total} = 682[1]	**< 26 years of age**	**Low income**
N (%)	554 (81%)	128 (19%)
Median age (IQR)	24 (22,25)	31 (28, 36)
N (%) with university degree	270 (49%)	98 (77%)
N (%) currently pursuing education	438 (80%)	57 (45%)
N (%) CH-Li as place of birth	397 (72%)	98 (77%)
N (%) assigned sex male	223 (40%)	58 (45%)
N (%) assigned sex female	329 (60%)	70 (55%)
N (%) gender identity male	217 (39%)	55 (43%)
N (%) gender identity female	305 (55%)	66 (52%)
N (%) gender identity other than male/female	23 (4%)	4 (3%)
N (%) no gender identity given	9 (2%)	3 (2%)
N (%) heterosexual	351 (63%)	74 (58%)
N (%) Bisexual	98 (18%)	21 (16%)
N (%) Homosexual (gay, lesbian)	48 (9%)	19 (15%)
N (%) Pansexual	29 (5%)	8 (6%)
N (%) use no terms to describe their sexuality	26 (5%)	5 (4%)
N (%) receiving diagnosis within pilot	27 (5%)	9 (7%)
N (%) Would not have tested without pilot project	196 (36%)	29 (23%)
N (%) would have tested less regularly	260 (48%)	75 (59%)

[1]Entries with age provided, CH-Li: Switzerland or Lichtenstein
