# Supplementary material for: Lowering the barriers to sexual health services: Impacts of free counselling and testing for sexually transmitted infections in Switzerland – an observational study
Source: PLoS One. 2026 Apr 1;21(4):e0327114. doi: 10.1371/journal.pone.0327114 (PMC13042815; doi:10.1371/journal.pone.0327114)
Supplement: S6 Table — 1Other: consisting of gender diverse people, women having exclusively sex with other women, and participants not using terms for either their gender or sexual orientation identity. 2 Emphasises specific sexual behaviour patterns or identity, transcending binary orientation categories. Abbreviations: 95% CI: 95% confidence interval for point estimate Lower being the lower limit and upper the upper limit of the 95% CI, HIV: human immunodeficiency virus, model: first test ~ low income + age + level of education + country of birth + demographic group, OR: odds ratio, STI: sexually transmitted infection. (PDF) [file pone.0327114.s006.pdf]

S6 Table: Odds ratios (95% CI) from logistic regression on first HIV/STI tests, FBQ data

				First HIV Test				First STI Test			
				OR	*95% CI*		*BIC*	*OR*	*95% CI*		*BIC*
**Variables**					*Lower*	*Upper*			*Lower*	*Upper*	
Reference group				**Sexual identity model[2] (N=670)**			817.44	**Sexual identity model[2] (N= 671)**			863.46
	N (HIV/STI)	***Qualified by***	***N (HIV/STI)***								
Age	546 / 543	Low income	124 / 128	0.81	0.37	1.72		0.71	0.36	1.40	
		Age		0.85	0.78	0.92		0.92	0.86	0.98	
		Education									
University degree	363 / 363	No university degree	307 / 308	0.96	0.66	1.38		1.01	0.71	1.42	
		Place of birth									
Switzerland	487 / 485	Other than Switzerland	183 / 184	1.43	0.98	2.09		1.03	0.75	1.60	
		Demographic group									
Women having sex with men	364 / 361	Men having exclusively sex with women	171 / 173	0.60	0.40	0.88		1.46	1.01	2.12	
		Men having sex with men	95 / 96	0.28	0.15	0.48		0.74	0.44	1.21	
		Other[1]	40 / 41	0.81	0.40	1.61		0.95	0.47	1.86	

[1]Other: consisting of gender diverse people, women having exclusively sex with other women, and participants not using terms for either their gender or sexual orientation identity. [2] Emphasises specific sexual behaviour patterns or identity, transcending binary orientation categories. **Abbreviations:** 95% CI: 95% confidence interval for point estimate Lower being the lower limit and upper the upper limit of the 95% CI, HIV: human immunodeficiency virus, model: first test ~ low income + age + level of education + country of birth + demographic group, OR: odds ratio, STI: sexually transmitted infection.
