# Supplementary material for: Lowering the barriers to sexual health services: Impacts of free counselling and testing for sexually transmitted infections in Switzerland – an observational study
Source: PLoS One. 2026 Apr 1;21(4):e0327114. doi: 10.1371/journal.pone.0327114 (PMC13042815; doi:10.1371/journal.pone.0327114)
Supplement: S7 Table — (PDF) [file pone.0327114.s007.pdf]

S7 Table: Likert-Matrix on the communication during the consultation as recorded in the feedback questionnaire (FBQ)

	Fully disagree	Somewhat disagree	Neither agree nor disagree	Somewhat agree	Fully agree
The HCP understood, what was concerning me.	0.7%	1.0%	1.4%	8.3%	88.6%
It was easy to trust the HCP.	0.6%	0.4%	2.2%	8.8%	88.0%
It was easy to ask questions.	0.4%	0.7%	1.8%	11.1%	86.0%
We had enough time to discuss all important topics.	0.4%	0.6%	1.7%	13.5%	83.9%
The discussion on my sexuality was appreciative.	0.6%	0.3%	6.5%	11.8%	80.9%
I understood why which tests were done.	0.6%	1.3%	0.8%	10.1%	87.3%
I consented to these tests.	0.6%	0.3%	0.4%	2.2%	96.5%
