# Supplementary material for: Lowering the barriers to sexual health services: Impacts of free counselling and testing for sexually transmitted infections in Switzerland – an observational study
Source: PLoS One. 2026 Apr 1;21(4):e0327114. doi: 10.1371/journal.pone.0327114 (PMC13042815; doi:10.1371/journal.pone.0327114)
Supplement: Attachment — Submitted filename: PONE-D-25-30038_Response to Reviewers.pdf [file pone.0327114.s009.pdf]

Point-by-point response to reviewers PONE-D-25-30038

Responses to Editors

We would like to thank the editors and reviewers for their careful reading of our manuscript and for the constructive and detailed feedback. We have addressed all comments point-by-point below and revised the manuscript accordingly. All line numbers refer to the revised manuscript (tracked changes).

Comment 1

1. Please ensure that your manuscript meets PLOS ONE's style requirements, including those for file naming. The PLOS ONE style templates can be found at

https://journals.plos.org/plosone/s/file?id=wjVg/PLOSOne_formatting_sample_main_body.pdf and

https://journals.plos.org/plosone/s/file?id=ba62/PLOSOne_formatting_sample_title_authors_affiliations.pdf.

> **Response to Comment 1**
> We thank the editors for the reminder regarding formatting and file-naming requirements. The requirements including file naming has been followed.

Comment 2

2. Thank you for stating in your Funding Statement:

"This work was supported by the City of Zurich (https://www.stadt-zuerich.ch/de.html), City Council Resolution GR Nr. 2021/432. Financial support from the Swiss Federal Office of Public Health (FOPH) (https://www.bag.admin.ch/bag/de/home.html) was received by the SwissPrEPared program and study. The funding organisations had neither any role in the design and conduction of the study; nor in the collection, management, analysis and interpretation of the data; nor in the preparation of the manuscript."

Please provide an amended statement that declares *all* the funding or sources of support (whether external or internal to your organization) received during this study, as detailed online in our guide for authors at http://journals.plos.org/plosone/s/submit-now. Please also include the statement "There was no additional external funding received for this study." in your updated Funding Statement.

Please include your amended Funding Statement within your cover letter. We will change the online submission form on your behalf.

Response to Comment 2

We appreciate the editors' guidance on the Funding Statement. The statement has been revised for completeness and clarity.

Revised Funding Statement

"Statement on funding sources: This work was supported by the City of Zurich (City Council Resolution GR Nr. 2021/432) and by the Swiss Federal Office of Public Health (FOPH) through support to the SwissPrEPared program and study. The authors did not receive any internal institutional funding specifically for this research. No funding, salary support, research materials, or any other contributions were received from Gilead, MSD, or ViiV Healthcare. The funding organizations had no role in the design or conduct of the study; in the collection, management, analysis, or interpretation of the data; or in the preparation of the manuscript. No commercial company provided funding or had any involvement in the study. There was no additional external funding received for this study."

---

3. Thank you for stating the following in the Competing Interests section:

"B.H. reports honoraria for advisory boards, lectures and travel grants paid to himself from the companies Gilead, MSD and ViiV, which are unrelated to the submitted work. J.S.F. reports grants for the institution from Gilead, MSD, and ViiV Healthcare, which are unrelated to the submitted work. The other authors declare no competing interests."

We note that one or more of the authors are employed by a commercial company: Gilead, MSD, and ViiV Healthcare

a. Please provide an amended Funding Statement declaring this commercial affiliation, as well as a statement regarding the Role of Funders in your study. If the funding organization did not play a role in the study design, data collection and analysis, decision to publish, or preparation of the manuscript and only provided financial support in the form of authors' salaries and/or research materials, please review your statements relating to the author contributions, and ensure you have specifically and accurately indicated the role(s) that these authors had in your study. You can update author roles in the Author Contributions section of the online submission form.

Please also include the following statement within your amended Funding Statement.

"The funder provided support in the form of salaries for authors [insert relevant initials], but did not have any additional role in the study design, data collection and analysis, decision to publish, or preparation of the manuscript. The specific roles of these authors are articulated in the 'author contributions' section."

If your commercial affiliation did play a role in your study, please state and explain this role within your updated Funding Statement.

b. Please also provide an updated Competing Interests Statement declaring this commercial affiliation along with any other relevant declarations relating to employment, consultancy, patents, products in development, or marketed products, etc.

Within your Competing Interests Statement, please confirm that this commercial affiliation does not alter your adherence to all PLOS ONE policies on sharing data and materials by including the following statement: "This does not alter our adherence to PLOS ONE policies on sharing data and materials." (as detailed online in our guide for authors http://journals.plos.org/plosone/s/competing-interests) . If this adherence statement is not accurate and there are restrictions on sharing of data and/or materials, please state these. Please note that we cannot proceed with consideration of your article until this information has been declared.

Please include both an updated Funding Statement and Competing Interests Statement in your cover letter. We will change the online submission form on your behalf.

Response to Comment 3

3a. Funding statement

We are grateful for the opportunity to clarify these points. None of the authors are employed by Gilead, MSD, or ViiV Healthcare, and these companies provided no funding, salary support, research materials, or any other contributions to this study. The research was funded solely by the City of Zurich and the Swiss Federal Office of Public Health (FOPH).

The commercial entities mentioned in the Competing Interests section had no role in the study design; data collection, management, analysis, or interpretation; manuscript preparation; or the decision to publish.

Our amended Funding Statement is printed under our Response to Comment 2.

Because no salaries or materials were provided by the companies listed in the Competing Interests, the required sentence ("The funder provided support in the form of salaries for authors…") does not apply.

3b. Competing Interest Statement

We thank the editors for this clarification. We confirm that none of the authors are employed by, or received salary support from, Gilead, MSD, or ViiV Healthcare. We have updated the Competing Interests Statement to make this explicit and included the required adherence sentence.

Revised Competing Interest Statement

"Statement on competing interests: B.H. reports honoraria for advisory boards, lectures, and travel grants paid to himself from Gilead, MSD, and ViiV Healthcare, which are unrelated to the submitted work. J.S.F. reports grants to the institution from Gilead, MSD, and ViiV Healthcare, which are unrelated to the submitted work. None of the authors are employed by these companies. The other authors declare no competing interests. This does not alter our adherence to PLOS ONE policies on sharing data and materials."

Comment 4

4. We note that you have indicated that there are restrictions to data sharing for this study. For studies involving human research participant data or other sensitive data, we encourage authors to share de-identified or anonymized data. However, when data cannot be publicly shared for ethical reasons, we allow authors to make their data sets available upon request. For information on unacceptable data access restrictions, please see http://journals.plos.org/plosone/s/data-availability#loc-unacceptable-data-access-restrictions.

Before we proceed with your manuscript, please address the following prompts:

a) If there are ethical or legal restrictions on sharing a de-identified data set, please explain them in detail (e.g., data contain potentially identifying or sensitive patient information, data are owned by a third-party organization, etc.) and who has imposed them (e.g., a Research Ethics Committee or Institutional Review Board, etc.). Please also provide contact information for a data access committee, ethics committee, or other institutional body to which data requests may be sent.

b) If there are no restrictions, please upload the minimal anonymized data set necessary to replicate your study findings to a stable, public repository and provide us with the relevant URLs, DOIs, or accession numbers. Please see http://www.bmj.com/content/340/bmj.c181.long for guidelines on how to de-identify and prepare clinical data for publication. For a list of recommended repositories, please see https://journals.plos.org/plosone/s/recommended-repositories. You also have the option of uploading the data as Supporting Information files, but we would recommend depositing data directly to a data repository if possible.

Please update your Data Availability statement in the submission form accordingly.

Response to comment 4

4a. Ethical, legal, and contractual restrictions

The study uses three types of data:

- Sensitive, individual-level cohort data governed by Swiss data protection law and the ethical approval from the Cantonal Ethics Committee Zurich (BASEC-Nr. 2018–02015). These data cannot be publicly shared because the legislation and ethics approval prohibit releasing identifiable or potentially re-identifiable participant information.
- Anonymous BERDA program data provided by Checkpoint Zürich and Test-In under a formal data-sharing agreement. Under this contract, the data remain the property of the providers (Checkpoint Zürich and Verein Sexuelle Gesundheit Zürich) and cannot be publicly shared or transferred to third parties without explicit written permission. These contractual obligations constitute a legal restriction on public deposition.
- Anonymous Feedback Questionnaires (FBQ) collected by the University of Zurich. During data collection, participants were informed that their

responses would be shared only in aggregated form. The Cantonal Ethics Committee Zurich granted exemption from Swiss Human Research Law (BASEC-Nr. Req-2023-00594) on the basis that the data are anonymous and disseminated only in aggregated form. For these reasons, the raw dataset cannot be publicly archived.

De-identified or aggregated data underlying the study results can be shared with qualified researchers upon reasonable request. All requests will be evaluated by the SwissPrEPared Scientific Board in consultation with the relevant ethics bodies and in accordance with Swiss legal, ethical, and contractual requirements.

4b. Updated Data Availability Statement
Our revised Data Availability Statement is provided below.
The updated statement reads:

The datasets analysed in this study include (i) sensitive, individual-level participant data from the SwissPrEPared cohort, (ii) anonymous BERDA program data provided by Checkpoint Zürich and Verein Sexuelle Gesundheit Zürich under a contractual data-sharing agreement and (iii) anonymous feedback questionnaire data collected by the University of Zurich. In accordance with Swiss data protection law, the conditions of ethical approval from the Cantonal Ethics Committee Zurich (BASEC-Nr. 2018–02015 and BASEC-Nr.: Req-2023-00594), and the contractual restrictions imposed by the data owners, these data cannot be made publicly available.

De-identified or aggregated data underlying the analyses may be made available to qualified researchers upon reasonable request. All requests will be reviewed by the SwissPrEPared Scientific Board and, where necessary, the relevant ethics bodies, and must comply with applicable Swiss legal, ethical, and contractual requirements.

Data access requests may be directed to:
SwissPrEPared Study Coordination Office
University of Zurich
Department of Public & Global Health
Hirschengraben 84
8001 Zurich, Switzerland
Email: info@swissprepared.ch

Comment 5

5. Thank you for stating the following in the Acknowledgments Section of your manuscript:

“This work was supported by the City of Zurich, City Council Resolution GR Nr. 2021/432. Financial support from the Swiss Federal Office of Public Health (FOPH) was received by the SwissPrEPared program and study. The funding organisations

had neither any role in the design and conduction of the study; nor in the collection, management, analysis and interpretation of the data; nor in the preparation of the manuscript."

We note that you have provided additional information within the Acknowledgements Section that is not currently declared in your Funding Statement. Please note that funding information should not appear in the Acknowledgments section or other areas of your manuscript. We will only publish funding information present in the Funding Statement section of the online submission form.

Please remove any funding-related text from the manuscript and let us know how you would like to update your Funding Statement. Currently, your Funding Statement reads as follows:

"This work was supported by the City of Zurich (https://www.stadt-zuerich.ch/de.html), City Council Resolution GR Nr. 2021/432. Financial support from the Swiss Federal Office of Public Health (FOPH) (https://www.bag.admin.ch/bag/de/home.html) was received by the SwissPrEPared program and study. The funding organisations had neither any role in the design and conduction of the study; nor in the collection, management, analysis and interpretation of the data; nor in the preparation of the manuscript."

Please include your amended statements within your cover letter; we will change the online submission form on your behalf.

Response to comment 5
We thank the editors for this clarification. All funding-related text has now been removed from the Acknowledgments section of the manuscript, as required. The Funding Statement has been updated and consolidated accordingly (see responses to comments 2 and 3), and it now contains the complete and accurate description of all funding sources. No funding information remains in the Acknowledgments section.

Comment 6
6. If the reviewer comments include a recommendation to cite specific previously published works, please review and evaluate these publications to determine whether they are relevant and should be cited. There is no requirement to cite these works unless the editor has indicated otherwise.

Response to comment 6
We appreciate this clarification. The reviewers did not suggest including specific previously published work.

Response to Reviewer 1

We thank Reviewer 1 for their thoughtful comments. Below we address each point in detail. Lines we refer to are from the tracked-changes manuscript.

Comment 1

This study describes the implementation of free STI counselling and testing for persons < 26 years and people with low income in Zurich, Switzerland. Routinely collected health data and a client feedback questionnaire were used to assess factors associated with first HIV/STI testing in the first 12 months of the program and examined potential barriers. The collected data set appears to be a representative sample of persons using the testing centers. The study is important as the result of this project may give important insights towards implementing free STI counselling and testing in other high income settings where barriers towards testing are different than in low/middle-income settings. The used statistical methods seem appropriate for the study.

My main concern is that research question 3 (impact on workload/capacitiy of the testing centers) cannot be adequately answered with the collected data as this is only evaluated with one question from the voluntary feedback questionnaire on wait times and the mention of the overall performed number of visits. However, there is no information on what the "usual" number of visits is for the testing centers, therefore as a reader no conclusion can be drawn whether this is an increase/additional burden or not. I would suggest to either adapt/remove research question 3 and/or either elaborate more on the limitation of the available data to answer this question or if this data is available include it in the manuscript.

> Thank you very much for pointing this out. We agree and hence rephrased the question to «(iii) the potential added burden on existing sexual healthcare services resulting from the implementation of free VCT.” (lines 90-91). And accordingly adapted the Results (lines 490 - 496) and Discussion (lines 519 - 520, 628 - 634). Moreover, we added a section to the Limitations (lines 671-677).

Comment 2

Background:
1. please include 1-2 sentences on the background of the HIV burden of key populations in Switzerland, so readers know on which populations need to be focused on/reach by testing strategies

> Thank you for the suggestion. We rewrote the background section and added a short paragraph on the HIV burden in Switzerland. (Lines 60-70).

Comment 3
Methods:
1. please specify in the methods section that the FBQ questionnaire was not sent to people who were tested during a PrEP visit

> Thank you very much for the comment. We added this restriction to the methods section (lines 233 - 234).

Comment 4
1. Line 159-160: please delete: (Error! Reference source not found.)

Thank you for pointing this out. For avoiding this in the future, all links and cross-references have been removed to avoid this issue in future.

Comment 4

2. Table 1: The table in the format I received is hard to read with all the numbers and line changes (often mid-word). Potentially a different format (horizontal), different font size and differing background colors could help to make it more readable and thus understandable. Consider doing two separate tables for characteristics/reasons for accessing VCT and the results

Thank you for this suggestion. We have split the table into two tables as you suggested and formatted them to be horizontal (Table 1 and Table 2).

Comment 5

3. Line 167 (+ table): Stage III/laten, please change to Stage III/latent (and a superscripted "e" instead of a "3" as all the other footnotes are letters and not numbers)

Thank you for spotting our typo and inconsistent superscript conventions. These errors have been corrected (Table 2).

Comment 6

4. Is there any information of the educational background in the general population taking up the free testing (not only from the ones completing the voluntary FBQ survey)? This might be interesting to see if in general persons with a higher education are more likely to take up free STI testing or if the bias mainly exists because they are more likely to fill out the survey.

Thank you for this comment. Education level was only systematically captured in the FBQ and in PrEP visits. It was not collected consistently in routine VCT records throughout the study period. Because education data were incomplete and not representative of the full VCT population, we did not include them in the main analyses.

Comment 7

5. Are the people coming for their first STI test the same as coming for their first HIV test or are these different groups? In the persons filling out the FBQ survey the difference between the two groups is only two (with two more first STI testing). I was not able to find the information of the overall study population, in the text only first HIV tests are mentioned. Is there a reason you are not reporting on first STI tests in the overall study population?

Thank you for this question. 1) All people coming to a VCT visit were invited to participate in the feedback questionnaire (FBQ). However, due to the anonymous nature of both the VCT records and the FBQ, we cannot link these two data sets. This prevents us from comparing first-time HIV testing and first-time STI testing across the entire study population.

2) Routine VCT documentation only captured whether the visit was a first HIV test. There was no equivalent variable indicating first STI test. Information on first-time STI testing was available only through the FBQ. Therefore, we were unable to determine first-time STI testing status for the full VCT population and could report it only for FBQ respondents.

Comment 8

Discussion:
- how do you explain the low number of tests during PrEP visits? Different population or is there a different way for subsidized testing

Thank you for this observation. The relatively low number of PrEP-related tests in our dataset reflects the structure and eligibility criteria of the free VCT programme rather than differences in testing behaviour. The programme primarily targets young people (<26 years) and people with low income. In Switzerland, PrEP users tend to be older (median age ~40 years in the national programme), highly educated, and financially stable, and therefore only a small proportion qualify for free VCT. The age of the Swiss PrEP cohort is comparable to similar cohorts in Europe. We have now discussed this point in the discussion (lines 598 - 611).

Response to Reviewer 2

We thank Reviewer 2 for the constructive feedback. We address each point in detail below. Lines refer to the tracked-changes version of the manuscript.

Comment 1

1. In the abstract you have mentioned that "MSM, were significantly less likely to be first-time testers for HIV (OR: 0.28, 95% CI: 0.15–0.48) and STI (OR: 0.74, 95% CI:0.44–1.21) compared to women who have sex with men (WSM)". The confidence interval crosses 1, so the results do not remain significant. Kindly look into it and address this in the abstracts as well as in your manuscript.

Thank you for spotting our imprecise wording. The Abstract text has been adapted to read:

«MSM were significantly less likely to be first-time testers for HIV (OR: 0.28, 95% CI: 0.15–0.48) and were also less likely to be first-time testers for STIs (OR: 0.74, 95% CI: 0.44–1.21) compared to women who have sex with men (WSM).” (lines 43 - 47)

Comment 2

2. The BACKGROUND seems not well structured. The starting line “For Switzerland to reach the next World Health Organization (WHO) goal of 95-95-95 in the HIV-cascade by 2025, the reach of its HIV testing programmes need to be expanded” is not impactful. The whole Background needs to be rewritten to create a compelling

narrative to capture the reader’s interest.

Thank you for the input. The background has been rewritten for improved structure and narrative coherence. It now follows this structure: Public health need – HIV epidemiology in Switzerland – VCT as potential solution – structural barriers to VCT – municipal intervention (lines 53 - 92).

Comment 3

3. The data set counts clinic visits, not named people, and 39% of them were first-time testers. Repeat attenders can bias estimates. Explicitly mentioning "visits as units of analysis" (not individuals) in methodology can clarify the methodology.
A sensitivity analysis where you can assume some plausible repeat rates (like 10%, 20%) and its effect on the main results can also be a valuable addition to the statistical measures.

Thank you for raising this important methodological point. We now clearly state in the Methods section that visits (and completed FBQs) were the units of analysis, as anonymous VCT procedures do not allow individuals to be identified across visits (lines 261 - 265).
Regarding the suggestion for a sensitivity analysis: because repeat visitors are, by definition, not first-time testers, these individuals should not bias the results describing visits for first-time testing even if they visit more than once. Our estimate of 39% therefore represents a conservative lower bound if you are discussing first-time testing on the individual level; the individual level proportion of first-time testers is likely higher. Moreover, due to the anonymous nature of the visits, we cannot readily identify repeated visits, hence, any assumed repeated-visits rate would be purely speculative. We expanded on this point in the limitations (lines 645 - 661)

We thank the editors and reviewers once again for their constructive and thoughtful feedback. We hope that the revisions have addressed all concerns and improved the clarity and quality of the manuscript. We would be grateful for your consideration of the revised version, and we remain available to provide any further information if needed.

Yours sincerely,

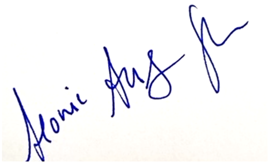

Leonie Arns-Glaser
Department of Public & Global Health
Epidemiology, Biostatistics and Prevention Institute
University of Zurich
Email: leonie.arns-glaser@uzh.ch
