# Supplementary material for: Lowering the barriers to sexual health services: Impacts of free counselling and testing for sexually transmitted infections in Switzerland – an observational study
Source: PLoS One. 2026 Apr 1;21(4):e0327114. doi: 10.1371/journal.pone.0327114 (PMC13042815; doi:10.1371/journal.pone.0327114)
Supplement: Attachment — Submitted filename: Response to Reviewers.docx [file pone.0327114.s010.docx]

**Point-by-point response to reviewers PONE-D-25-30038**

**Responses to Editors**

We would like to thank the editors and reviewers for their careful reassessment of our manuscript and our responses to their comments. Below follow the point-by-point answers to the remaining comments.

**Comment 1**
Please review your reference list to ensure that it is complete and correct. If you have cited papers that have been retracted, please include the rationale for doing so in the manuscript text, or remove these references and replace them with relevant current references. Any changes to the reference list should be mentioned in the rebuttal letter that accompanies your revised manuscript. If you need to cite a retracted article, indicate the article’s retracted status in the References list and also include a citation and full reference for the retraction notice.

**Response to comment 1**
We thank the editor for the reminder to check our references. In the reference below, there was a wrong DOI number. This has been rectified in the manuscript.

Mitjà O, Padovese V, Folch C, Rossoni I, Marks M, Arias MAR, et al. Epidemiology and determinants of reemerging bacterial sexually transmitted infections (STIs) and emerging STIs in Europe. Lancet Reg Health Eur. 2023;34: 100778. doi:10.1016/j.lanepe.2023.100742

Moreover, the CDC has moved one of the referenced resources. The link has been updated in the reference list:

Centers for Disease Control and Prevention (CDC). Trends in the prevalence of sexual behaviors and HIV testing: National YRBS 1991–2019 [Internet]. 2021 [cited 2026 February 26]. Available from: https://www.cdc.gov/yrbs/?CDC_AAref_Val=https://www.cdc.gov/healthyyouth/data/yrbs/factsheets/2019_sexual_trend_yrbs.htm

**Additional Editor Comments:**

Kindly address a minor comment from reviewer 2. Thank you

**Response to additional Editor Comments**
Thank you very much for reminding us of reviewer 2’s comment. We have implemented the change in the abstract to fit with their comment and replied to it below.

**Response to Reviewers**

We sincerely thank both reviewers for the time and efforts in reassessing our manuscript.

**Comment Reviewer 2**

You mentioned in the abstract that "MSM were also less likely to be first-time testers for STIs (OR: 0.74, 95% CI: 0.44–1.21) compared to women who have sex with men (WSM)" is not clarified properly. Since, the CI exceeds value of 1, the relation should be reported a "non-significant", "less likely" is rather misleading. Kindly correct it.

**Response to Comment Reviewer 2**

Thank you very much for pointing out the phrase that could be misunderstood. We now explicitly point out the non-significance of the second association:

“MSM were significantly less likely to be first-time testers for HIV (OR: 0.28, 95% CI: 0.15–0.48) and also less likely to be first-time testers for STIs (OR: 0.74, 95% CI: 0.44–1.21) compared to women who have sex with men (WSM); however, the latter association was non-significant.”

We thank the editors and reviewers once again for their constructive and thoughtful feedback improving our manuscript. We would be grateful for your consideration of the revised version, and we remain available to provide any further information if needed.

Yours sincerely,

Leonie Arns-Glaser

Department of Public & Global Health

Epidemiology, Biostatistics and Prevention Institute

University of Zurich

Email: leonie.arns-glaser@uzh.ch
